# Scalable Production of Ambient Stable Hybrid Bismuth‐Based Materials: AACVD of Phenethylammonium Bismuth Iodide Films**

**DOI:** 10.1002/chem.202100774

**Published:** 2021-05-27

**Authors:** M. Wang, C. Sanchez‐Perez, F. Habib, M. O. Blunt, C. J. Carmalt

**Affiliations:** ^1^ Department of Chemistry University College London 20 Gordon Street London WC1H 0AJ UK; ^2^ Department of Telecommunications Engineering Instituto de Energía Solar Universidad Politécnica de Madrid Avenida Complutense s/n 28040 Madrid Spain

**Keywords:** lead-free hybrid material, light-harvesting, phenethylammonium bismuth iodide, scalability

## Abstract

Large homogeneous and adherent coatings of phenethylammonium bismuth iodide were produced using the cost‐effective and scalable aerosol‐assisted chemical vapor deposition (AACVD) methodology. The film morphology was found to depend on the deposition conditions and substrates, resulting in different optical properties to those reported from their spin‐coated counterparts. Optoelectronic characterization revealed band bending effects occurring between the hybrid material and semiconducting substrates (TiO_2_ and FTO) due to heterojunction formation, and the optical bandgap of the hybrid material was calculated from UV‐visible and PL spectrometry to be 2.05 eV. Maximum values for hydrophobicity and crystallographic preferential orientation were observed for films deposited on FTO/glass substrates, closely followed by values from films deposited on TiO_2_/glass substrates.

## Graphical abstract

**Compact and stable** bismuth halide‐based hybrid material thin films were grown using aerosol‐assisted chemical vapor deposition. The dependence of structural and optoelectronic properties of phenethylammonium bismuth iodide films were evaluated on varying substrates, deposition temperatures and curing conditions. The morphology and optical band‐bending effects were related to synthetic parameters, and crack‐free films were obtained with high moisture resistance.

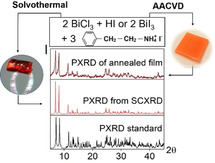

## Introduction

**Figure 1:**
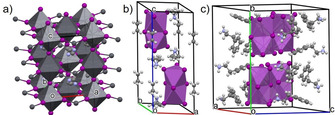
Unit cell of (a) CH_3_NH_3_PbI_3_ ^[35]^ (b) [CH_3_NH_3_]_3_[Bi_2_I_9_]^[33]^ and (c) [C_6_H_5_(CH_3_)_2_NH_3_]_3_[Bi_2_I_9_] (or [PEA]_3_[Bi_2_I_9_]) reproduced using Mercury®.

Organic‐inorganic hybrid perovskites and perovskite‐derived materials have shown unique photo‐ and electro‐chemical properties, which derive from their high defect‐tolerance,^[1]^ well‐balanced charge transfer,^[2]^ high optical absorption coefficient^[3]^ and long‐range electron‐hole diffusion length.^[4]^ Lead halide‐based perovskites, such as methylammonium lead iodide (CH_3_NH_3_PbI_3_), exhibit premier semiconducting properties for optoelectronic applications, such as solar cells,^[5]^ energy storage devices^[6]^ and photocatalysis.^[7]^ In just one decade, the power conversion efficiencies (PCE) of these third generation perovskite solar cells (PSCs) have rapidly increased from 3.81 % to 25.2 %,[^5^, ^8^] which is comparable to the efficiency of industrially produced single crystalline silicon solar cells.^[9]^ However, despite their great performance, CH_3_NH_3_PbI_3_‐based solar cells suffer from intrinsic drawbacks that hinder their commercialization, including low chemical instability in ambient conditions, low thermal stability,[^10^, ^11^] current‐voltage hysteresis^[12]^ and the high toxicity of lead‐containing decomposition subproducts.^[13]^ Furthermore, alkylammonium cations like CH_3_NH_3_^+^ are markedly hydroscopic, which makes CH_3_NH_3_PbI_3_ liable to decompose irreversibly in humid ambient conditions and ultimately deteriorates device performance.[^11^, ^14^] In fact, upon continuous exposure to moisture, it decomposes into CH_3_NH_3_I, PbI_2_ and Pb^(0)^,[^11^, ^14^, ^15^, ^16^] and so the research of alternative environmentally friendly lead‐free perovskite‐derived materials with high air/humidity stability and at least comparable conversion efficiencies is in high demand. Although some alternative metals like tin,^[17]^ germanium^[18]^ and copper^[19]^ have been investigated to substitute lead in the structure, bismuth is considered the best candidate since it has a similar electron configuration and ionic radius to lead,^[20]^ and forms materials with high ambient stability, tunable bandgaps and good solution processability.[^21^, ^22^, ^23^, ^24^, ^25^, ^26^, ^27^] Much research has been carried out around methylammonium bismuth iodide ([CH_3_NH_3_]_3_[Bi_2_I_9_])[^25^, ^28^, ^29^, ^30^] solar cells, however although over a 60‐fold improvement in chemical stability has been achieved in comparison to CH_3_NH_3_PbI_3_‐based PSCs,[^20^, ^31^] their low conversion efficiencies (3.17 %)^[30]^ hinder potential use in photovoltaics. The main reasons detected for such low device efficiency are the existence of non‐radiative recombination induced by defect states in the bandgap, morphological flaws and the associated low carrier transport across interfaces caused by the fast crystallization of the [CH_3_NH_3_]_3_[Bi_2_I_9_] films.^[32]^ Film morphology is directly related to the recombination rate of photogenerated charges, and therefore high roughness, cracks and pin‐holes are detrimental towards the PCE of the final solar cell assembly.^[23]^ A substantial intrinsic difference between lead and bismuth‐based hybrid materials lies within their crystal structures: while CH_3_NH_3_PbI_3_ has 3D structure of corner‐sharing octahedra (perovskite structure, Figure 1a),^[13]^ [CH_3_NH_3_]_3_[Bi_2_I_9_] exhibit a derived 0D array of disconnected face‐sharing octahedra^[33]^ (Figure 1b). These structural differences are thought to be the cause of the lower absorption coefficient and the uniquely low thermal conductivity of [CH_3_NH_3_]_3_[Bi_2_I_9_], otherwise a key feature for the design of thermoelectric devices.[^32^, ^34^] To fully understand design limitations of PSCs, a better understanding of the underlying structure‐property relationships is still necessary, hence fabrication methods that yield defect‐free, compact and homogeneous films should be developed.^[27]^

Varied approaches such as cation displacement,^[36]^ vapor assisted solution process^[30]^ and anti‐solvent assisted crystallization solution process^[37]^ have successfully managed to improve film coverage and achieve a smooth morphology. Growth of films with preferential orientation and high thermal stability has been attained replacing the organic fragments with metallic cations,[^38^, ^39^] and large hydrophobic organic cations such as formamidinium or phenethylammonium (C_6_H_5_(CH_3_)_2_NH_3_^+^ or PEA^+^) have shown enhanced resistivity against moisture.[^23^, ^40^, ^41^] The steric effect and ambipolar nature of the large organic ligand is useful in passivating the interface between perovskite and hole/electron transport layers in PSCs,[^42^, ^43^, ^44^, ^45^] which not only improves the device stability, but also PCE *via* reduction of the nonradiative loss due to suppression of defect formation and achievement of low levels of self‐doping.[^23^, ^46^] The existence of aromatic cations causes quantum confinement in the perovskite materials, band gap widening and resulting in deposited films with different colors.^[47]^ Even though efficiencies have not matched those of lead‐based PSCs, the relatively large bandgap and high stability of bismuth hybrid materials with bulky organic ligands, such as phenethylammonium bismuth iodide or [PEA]_3_[Bi_2_I_9_] (Figure 1c), make them ideal candidates for tandem solar cells, acting as wide‐band‐gap top cell in combination with lower bandgap semiconductors for example silicon, CIGS/Se or perovskites.[^23^, ^48^] Furthermore, their high resistivity and thermal stability make them potential candidates as high‐performance X‐ray detectors,[^49^, ^50^] and in many other cutting‐edge applications such as photosensing and aqueous battery systems,[^51^, ^52^] making their further development extremely interesting. Despite having a large potential span of applicability, the lack of a scalable and cost‐effective synthetic process for this class of materials represents a large liability for their development and commercialization. Hybrid perovskite thin films are typically prepared by spin‐coating, which not only yields films with very small crystallite sizes, but its production is often limited to the use of a glovebox. In addition, while spray coating has been utilized on large‐scale substrates, excessive variables in the coating process such as droplet size, substrate wettability and solvent boiling point make it challenging,[^53^, ^54^] as well as crystallization conditions for materials with large organic cations.[^47^, ^55^] Solvent‐free techniques such as thermal co‐evaporation and high‐low vacuum deposition can produce high quality and pin‐hole free films with large crystallites due to achievement of a slower rate, however the demand of high vacuum makes them energy consuming and pricey,[^22^, ^56^] and high deposition temperatures in vacuum‐based methods have a high requirement on thermal stability of precursors.^[57]^ The need for a simple and low‐cost method to produce thin films of hybrid perovskite or perovskite‐derived materials requires the development of solution‐based methods beyond spin‐coating or spray coating. Aerosol‐assisted chemical vapor deposition (AACVD) represents an attractive alternative, as its solution‐based methodology adds versatility to the CVD process allowing the use of relatively large organometallic precursors carried in small droplets by a stream of inert gas.^[58]^ This method simplifies overall synthetic instrumentation and eliminates the necessity of a glovebox for the synthetic process. In addition, it allows deposition over large substrates of different nature and is compatible with large‐scale production. Hybrid lead halide perovskite films have been fabricated *via* AACVD and applied in PSCs successfully,[^54^, ^59^, ^60^, ^61^, ^62^] but fabrication of bismuth‐based materials using AACVD has not been reported yet due to fewer studies on bismuth‐based film growth. In this study uniform and dense thin films of [PEA]_3_[Bi_2_I_9_] with different morphologies were synthesized using AACVD over three types of substrates (glass, TiO_2_ and FTO) and their optoelectrical properties were investigated. This study offers a cost‐effective and scalable way to prepare moisture resistant bismuth‐based hybrid films and establishes AACVD as a promising strategy to address the large‐scale manufacturing hybrid lead‐free coatings.

## Results and Discussion

**Figure 2:**
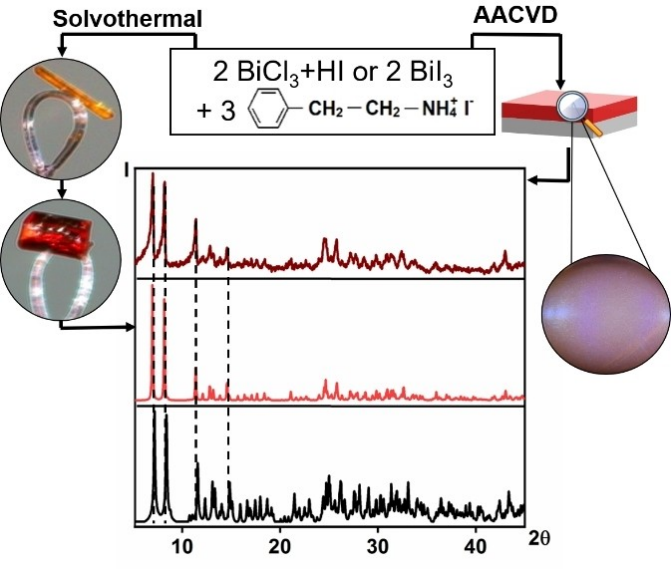
Synthesis methodologies employed to obtain [PEA]_3_[Bi_2_I_9_] single crystals and film coatings. PXRD patterns of scraped annealed film on glass (top) and calculated from SCXRD data (middle) are consistent with literature data (bottom).^[23]^

Orange needle‐shaped [PEA]_3_[Bi_2_I_9_] crystals were obtained from the solvothermal synthesis of BiCl_3_ and phenethylammonium iodide in methanol at 140 °C for 24 h. Strongly diffracting red block‐shaped crystals were collected after recrystallisation from CH_3_NO_2_. Crystallographic data of the recrystallized sample was consistent with literature,^[23]^ confirming the chemical identity of [PEA]_3_[Bi_2_I_9_] (Figure 1c, Table S1). Data modelled from the single crystal X‐ray diffraction (SCXRD) standard of the crystals of [PEA]_3_[Bi_2_I_9_], using Diamond® software, was then used to compare to the powder XRD (PXRD) pattern obtained from the film deposition. Thus, AACVD of BiI_3_ and phenethylammonium iodide in DMF was investigated to deposit [PEA]_3_[Bi_2_I_9_] thin films. The PXRD pattern obtained from delamination of an annealed [PEA]_3_[Bi_2_I_9_] thin film agreed with data modelled from the SCXRD standard (Figure 2), proving the ability of AACVD to deposit crystalline coatings of bismuth‐based hybrid materials.

**Figure 3:**
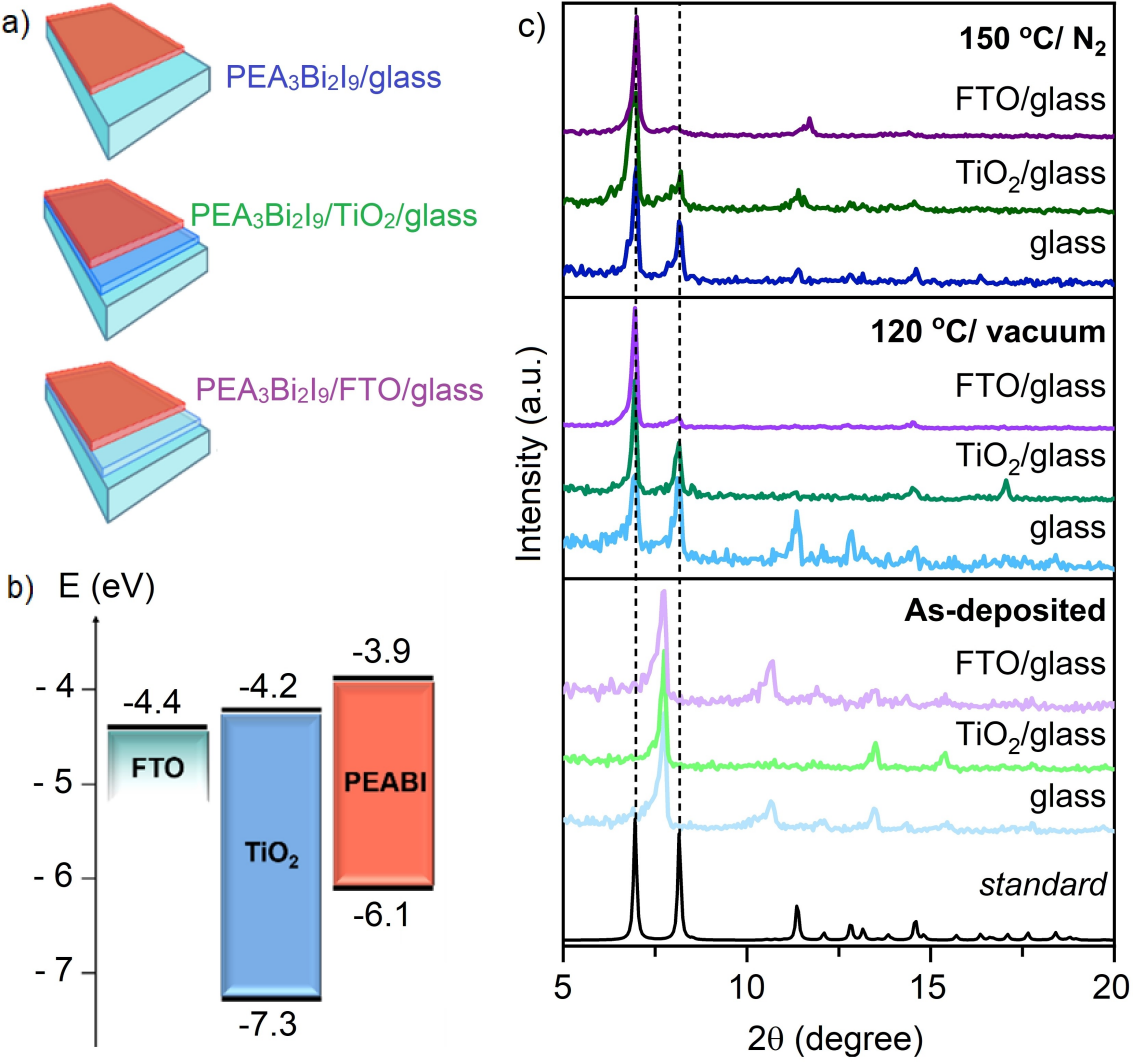
(a) Scheme of [PEA]_3_[Bi_2_I_9_] films deposited on different substrates, (b) Energy alignment diagram with respect to the vacuum level of [PEA]_3_[Bi_2_I_9_], TiO_2_ and FTO^[63]^ and (c) GIXRD patterns of [PEA]_3_[Bi_2_I_9_] films deposited on float glass (blue), TiO_2_/glass (green) and FTO/glass (purple) as‐deposited, vacuum annealed (120 °C) and annealed in N_2_ (150 °C), in comparison to [PEA]_3_[Bi_2_I_9_] SCXRD.

[PEA]_3_[Bi_2_I_9_] thin films were deposited on three different substrates: float glass, AACVD‐deposited TiO_2_ on float glass and FTO on float glass (Figure 3a). Deposition on glass was pursued to investigate the effect of an amorphous substrate on the film growth mechanism, and to provide optical data without band‐bending interferences. The metal oxides were selected as substrates based on their favorable energy alignment with [PEA]_3_[Bi_2_I_9_] (Figure 3b).^[63]^ Deposition temperatures from 100 to 200 °C in steps of 25° were tested in the AACVD process.

**Figure 4:**
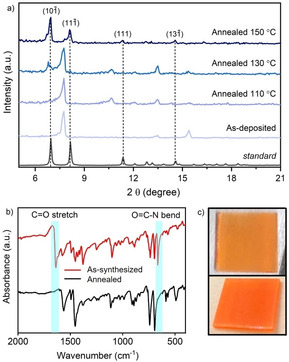
(a) GIXRD patterns of [PEA]_3_[Bi_2_I_9_] films deposited on glass and annealed in N_2_ at different temperatures in comparison to [PEA]_3_[Bi_2_I_9_] SCXRD standard; (b) FT‐IR spectra of as‐deposited and annealed in N_2_ (150 °C) [PEA]_3_[Bi_2_I_9_] thin films and (c) images of as‐deposited (top) and annealed in N_2_ (150 °C) (bottom) [PEA]_3_[Bi_2_I_9_] coatings on glass.

**Figure 5:**
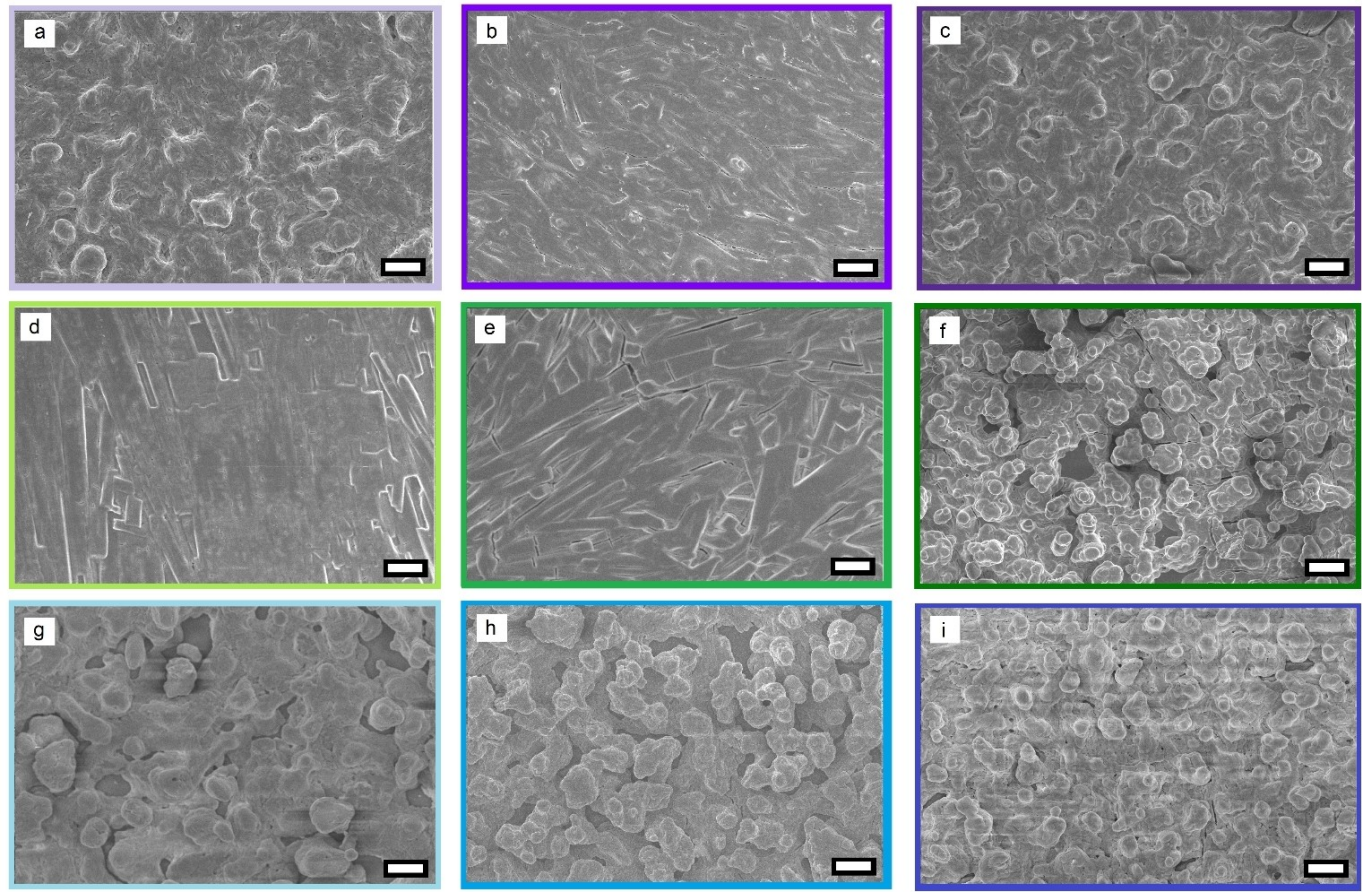
SEM images of hybrid thin films as‐deposited (left column), vacuum annealed at 120 °C (middle column) and annealed in N_2_ at 150 °C (right column) on FTO/glass substrates (purple, a–c), TiO_2_/glass (green, d‐f) and float glass (blue, g‐i). Magnification for all images is ×1000 and scale bar represents 10 μm.

Total coverage of the substrates with robust and adherent films was achieved at 125 °C, and lower or higher deposition temperatures promoted the formation of patchy and powdery films. Grazing angle (GI)XRD patterns of the as‐deposited films exhibited a slight shift of all reflections towards lower 2θ values when compared to the standard from the PXRD, which is related to a small cell expansion due to the co‐crystallized solvent molecule, DMF (Figure 3c).^[64]^ Thin films deposited on FTO/glass and annealed showed the strongest preferential orientation towards the (10$\bar{1}$ ) plane (2θ=6.92°). While pure‐phase materials were obtained at 120 °C in vacuum conditions, higher temperature was required to fully evaporate DMF from the structure (Figure 4a) when films were annealed in N_2_. The FT‐IR spectrum of the as‐deposited [PEA]_3_[Bi_2_I_9_] film on glass showed DMF also (Figure 4b), with peaks consistent with the presence of DMF in the as‐deposited film (1640 cm^−1^ {C=O stretch} and 663 cm^−1^ {O=C‐N bend}).^[65]^ Similar DMF inclusion hybrid inorganic‐organic films have previously been detected for AACVD and spin‐coated coatings synthesized using DMF,[^60^, ^66^] due to the higher boiling point of DMF than commonly used deposition temperatures.^[67]^ DMF can also form adducts with BiI_3_,^[68]^ further supporting the possibility of structural incorporation. Therefore, films were subsequently annealed for 30 minutes either under N_2_ or in a vacuum oven. Anneal processes led to removal of co‐crystallized DMF to form pure‐phase coatings, increasing their color intensity (Figure 4c). The composition and oxidation state of the elements present in a pure‐phase film deposited on glass and annealed at 150 °C in N_2_ were characterized using X‐ray photoelectron spectroscopy (XPS) and energy dispersive spectroscopy (EDS). Deconvolution of the C1s signal was best fitted using 2 components at 284.8 and 286.1 eV, fitting the respective presence of C−C and C−N environments in the PEA^+^ fragment (Figure S1).[^44^, ^69^] The N 1s environment had 2 components found at 402.4 and 400.7 eV, respectively. The first peak can be attributed to nitrogen in alkylammonium environment in [PEA]_3_[Bi_2_I_9_],^[70]^ and the second peak can be associated to adsorbed CH_3_NH_2_,^[60]^ likely produced as a dissociation product of phenethylammonium iodide. The Bi 4 f and I 3d spectra were best fitted to an environment with 2 components (Bi 4f_7/2_=159.6 eV and Bi 4f_5/2_=164.9 eV; I 3d_5/2_=619.8 eV and I 3d_3/2_=631.3 eV), corresponding to the characteristic signals of Bi^3+^ and I^−^.^[71]^ Additionally, a single environment for oxygen was detected (O 1s=532.7 eV), assigned to the silica of the substrate,^[70]^ and Si was observed in the survey spectrum as well (Figure S2). Bismuth and iodine were detected by EDS, with a ratio of Bi : I of ∼4.4, which agrees with the calculated value of 4.5 for [PEA]_3_[Bi_2_I_9_] and further confirming the chemical nature of the AACVD deposited thin film (Figure S3). Scanning electron microscope (SEM) analysis of the films showed significantly different growth trends and surface morphologies for each substrate and annealing condition set (Figure 5). Thin films deposited on plain glass were powdery with poor adhesion to the substrate and consisted of 5–10 μm sized particles. In contrast, uniform and compact films were formed with excellent coverage and adhesion when deposited on TiO_2_ and FTO layers, as would be expected using crystalline substrates.^[72]^ It is worth mentioning that while all films exhibit preferential orientation towards the (10$\bar{1}$ ) plane, flat surface crystallites were only detected in samples in which no contribution of the perpendicular (111) reflection (2θ=11.36°) was detected (Figure 3c), rendering shallow surfaces of flat crystallites in SEM (Figure 5).

**Figure 6:**
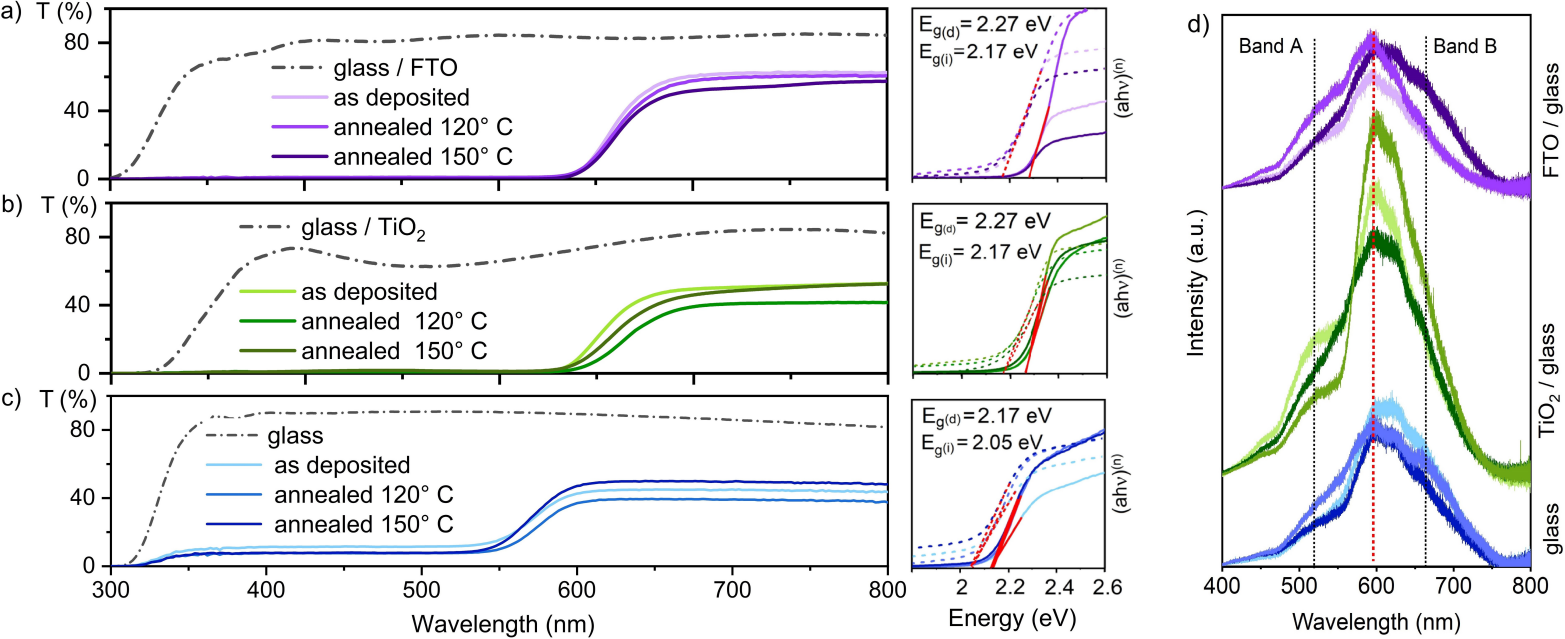
Transmittance spectrum of hybrid films on (a) FTO/glass, (b) TiO_2_/glass and (c) float glass. Direct and indirect optical bandgaps were calculated using the Tauc‐plot method where in (ahv)^(n)^ n=2 for direct bandgap (full lines) and n=1/2 for indirect bandgap (broken lines) calculations; (d) PL spectra of samples deposited on FTO/glass (top), TiO_2_/glass (middle) and float glass (bottom). Wannier‐Mott exciton binding energy is found at 599 nm (red dotted line).

Films deposited on TiO_2_ featured large and flat particles (5×60–100 μm) while those on FTO were small and block‐shaped (2×2 μm). For samples deposited over a metal oxide layer (TiO_2_ or FTO), annealing in vacuum conditions produced compact and adherent films with flat rectangular surface crystals. Cracks appeared in the surface of the film deposited on TiO_2_/glass, likely due to evaporation of DMF under vacuum, and no clear sintering process was evident (Figure 5e). Vacuum annealing of the film deposited on FTO/glass undergo sintering to form a compact and relatively smoother film (RMS roughness=0.128 μm) than reported perovskite films deposited *via* AACVD,^[54]^ which is shown in cross‐sectional SEM view (Figure S4) and AFM images (Figure S5). In particular the hybrid films deposited over FTO/glass have minimal grain boundaries^[73]^ and a crack‐less surface of as large as 4×20 μm crystals. The large preferential orientation towards a single crystallographic plane (10$\bar{1}$ ) observed for this sample (Figure 3c) is consistent with the formation of compact films with shallow surface features (Figure 5b). Annealing of samples in N_2_ atmosphere at 150 °C enabled development of high energy surfaces, and therefore results in particle agglomeration (5–10 μm) and surface roughening regardless of substrates (Figure 5). Contrary to previous reports,^[74]^ uniform thin films grown on different substrates under the same conditions show similar thicknesses (5–8 μm, Figure S4). Differences in crystal growth from spin‐coating and AACVD would explain discrepancies in thickness dependence,^[75]^ crystal size and preferred orientation.^[23]^ Optical characterization of the [PEA]_3_[Bi_2_I_9_] thin films deposited on all substrates was carried out through UV‐visible and PL spectra, and their optical bandgaps were calculated using the Tauc‐plot method^[76]^ (Figure 6a–c). The calculated indirect bandgaps of annealed films on TiO_2_/glass and FTO/glass (2.17 eV) (Figure 6a, b) were comparable to the values reported from [PEA]_3_[Bi_2_I_9_] films spin‐coated on FTO/glass (2.23 eV).^[23]^ Films prepared *via* AACVD (∼6 μm) were thicker than films reported using other methods (e. g. spin‐coating gives ∼300–600 nm),^[77]^ as shown in Figure S4, and therefore film transmittance (< 60 %) was significantly lower than in spin‐coated films (∼80 %).[^23^, ^77^] Low‐dimensional hybrid materials exhibit rather weak photoluminescence, hence weak intensities are expected. Wannier‐Mott excitation binding energies were estimated from the PL spectra (Figure 6d). The indirect bandgap obtained for films deposited on glass (2.05 eV) was in good agreement to that predicted from DFT calculations.^[23]^ The difference in bandgaps is likely produced by band‐bending effects between [PEA]_3_[Bi_2_I_9_] and the semiconductor, TiO_2_ or FTO, which can change the work function of the material.^[78]^ This effect was also observed in CH_3_NH_3_PbI_3_ films, as their reported bandgaps shift between 1.8–2.2 eV when deposited on different substrates, such as glass, TiO_2_, FTO and ITO.[^20^, ^29^, ^32^, ^37^, ^71^, ^79^] It has been reported that 2D perovskite hybrid layers exhibit multiple emission peaks due to the formation of secondary phases.[^80^, ^81^] A stable λ_max_=599 nm is found for all samples (Figure 6d) and two additional excitation bands appear on the PL spectra of samples at roughly 525 and 658 nm (Figure 6d, bands A and B). The appearance of two similar bands has been previously reported in phenethylammonium iodide‐treated CH_3_NH_3_PbI_3_ ^[82]^ and variable contribution from each emission appears closely related to crystalline orientation: stronger contribution of 525 nm excitation appears in samples with visibly flat surface crystallites (Figure 5b, 5d and 5e) in which no contribution of (111) crystallographic plane was detected in the GIXRD patterns (Figure 3c).

**Figure 7:**
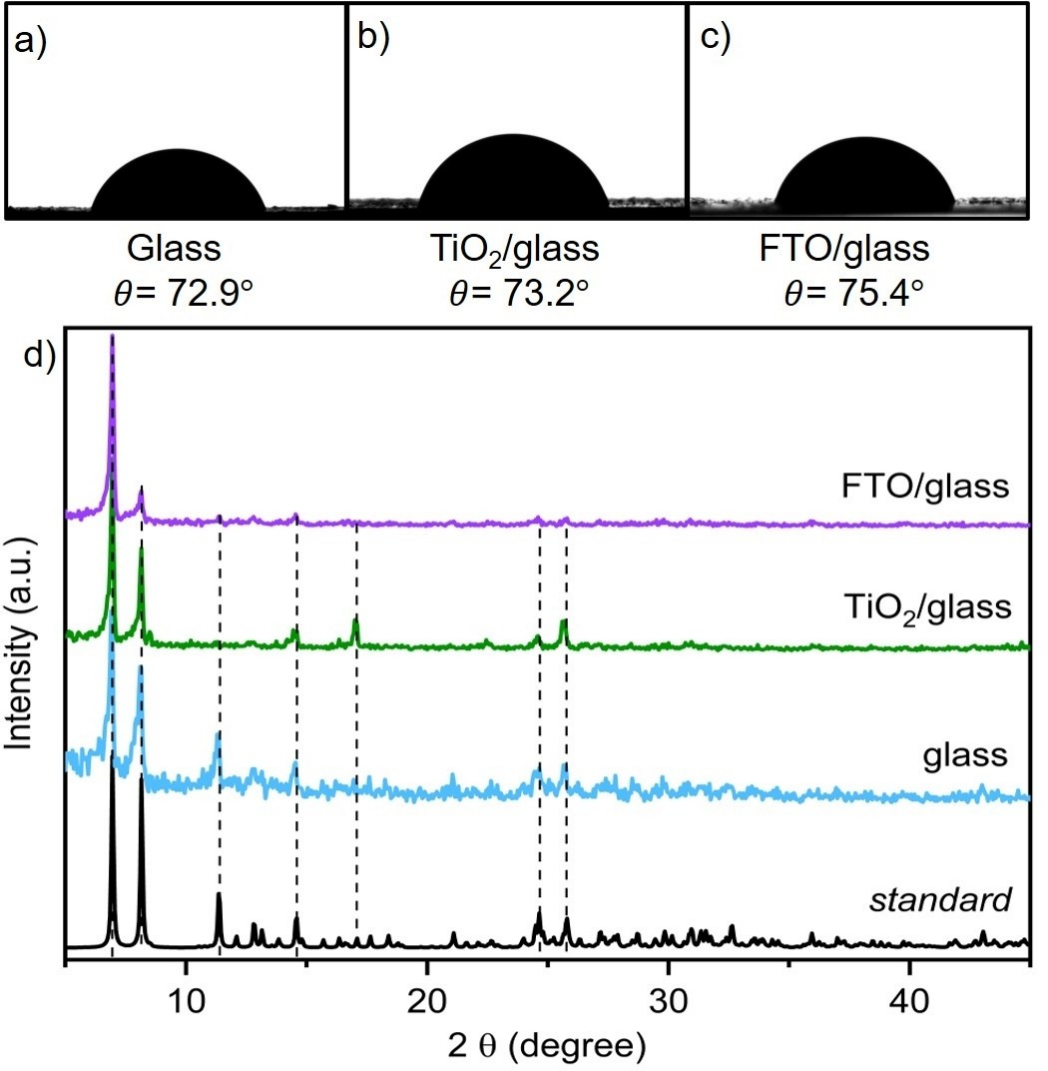
Water contact angles on vacuum annealed (120 °C) [PEA]_3_[Bi_2_I_9_] thin films deposited over (a) float glass, (b) TiO_2_/glass and (c) FTO/glass. (d) GIXRD patterns of vacuum annealed (120 °C) [PEA]_3_[Bi_2_I_9_] thin films deposited over float glass (blue), TiO_2_/glass (green) and FTO/glass (purple) after 6 months exposed to ambient conditions.

As crystal orientation on hybrid perovskite‐like materials has been confirmed to play a role on PL,^[83]^ such emissions are likely to appear due to changes in the population of surface traps. Therefore, trap‐mediated recombination is a possible reason for the appearance of different peak intensities.[^84^, ^85^] Since PL is a surface technique, band‐bending due to the formation of heterojunctions does not affect the measurement,^[86]^ and the bandgap of [PEA]_3_[Bi_2_I_9_] can be extracted without interfering factors. Contact angles of DMF on glass, TiO_2_/glass and FTO/glass substrates were found to be 45.8°, 23.7° and 12.9°, respectively (Figure S6). Low solvent contact angles are beneficial to reduce the nucleation energy barrier for film growth on a substrate,^[73]^ hence nucleation and growth of [PEA]_3_[Bi_2_I_9_] films from DMF solutions on FTO/glass substrates should be favored. The low contact angle of DMF on FTO also explains the strong preferential orientation observed in these films. Considering Neumann's equation cos(θ)=−1+2(γ_SV_/γ_LV_)^1/2^ ⋅ exp(‐β(γ_LV_‐γ_SV_)^2^),[^87^, ^88^] the solid surface tension is calculated to be 37.3 mJ m^−2^ for the [PEA]_3_[Bi_2_I_9_] film on FTO, which is substantially lower than those reported of 3D perovskite films,[^44^, ^88^] implying a better moisture resistance of [PEA]_3_[Bi_2_I_9_] films. In addition, the contact angle values of deionized water on films were tested to evaluate the hydrophilicity of films. Compared to values of lead‐based perovskite films,[^44^, ^89^] larger contact angles were detected for all films, namely 72.9° for films on glass, 73.2° for films on TiO_2_/glass and 75.4° for films on FTO/glass (Figure 7a–c), which represents a substantial improvement in water repellency.

Films on the three substrates were stored in ambient atmosphere up to 6 months, and no extra peaks of degradation or oxidized impurity was observed in XRD patterns (Figure 7d), which suggests that [PEA]_3_[Bi_2_I_9_] films *via* AACVD have a remarkable ambient stability. The overall improved water repellency of hybrid bismuth‐based films when using large aromatic cations like PEA^+^ is likely to provide longevity to relative devices under a humid environment, providing a door to their use in water‐based systems for energy production.

## Conclusion

Large coatings of lead‐free hybrid material [PEA]_3_[Bi_2_I_9_] were homogeneously formed over glass, TiO_2_/glass and FTO/glass substrates using AACVD coupled with a fast‐annealing process, requiring minimal preparation equipment and mild synthetic conditions. Films deposited on glass were powdery, however [PEA]_3_[Bi_2_I_9_] coatings deposited on metal oxide/glass substrates were adherent, compact, and exhibited a relatively smooth surface of large flat rectangular crystals with low RMS. Film thickness ranged from 5 to 8 μm and exhibited crystallographic preferential orientation when grown over a metal oxide, particularly in the case of FTO. Their chemical identity was confirmed through SCXRD, GIXRD, FTIR, XPS and EDS. Opto‐electronic analysis of the coatings revealed a strong influence of each substrate and annealing process. The optical bandgap of the [PEA]_3_[Bi_2_I_9_] films was found to be 2.05 eV, unlike the previously reported value, 2.23 eV. Band bending effects were observed in films deposited over semiconducting metal oxides/glass substrates due to the heterojunction formation between the two semiconductors. This work provides insight into the impact of substrates and film processing choice for potential large‐scale film production of organic–inorganic hybrid bismuth‐based materials. As a promising lead‐free hydrophobic material compatible with other hybrid perovskite coatings, AACVD‐grown [PEA]_3_[Bi_2_I_9_] coatings exhibit great stability against moisture and tunable morphologies. The work described herein is the first report on bismuth hybrid thin film synthesis using AACVD. As such, the results of our study set the foundation for the longed‐for transition to industrial‐scale fabrication of organic–inorganic hybrid lead‐free materials.

## Experimental Section

**Synthesis of [PEA]_3_[Bi_2_I_9_] single crystals**: [PEA]_3_[Bi_2_I_9_] polycrystalline samples were prepared *via* solvothermal synthesis. BiCl_3_ (1.00 mmol, 0.315 g, Alfa Aesar) and phenethylammonium iodide (0.75 mmol, 0.170 g, Sigma‐Aldrich) were dissolved in 12 mL methanol (Fisher Chemical) (each). The two solutions were mixed with additional 5 mL HI (57 wt %) and allowed to stir at room temperature for 15 min. The final solution was transferred into in a Teflon‐lined stainless‐steel 50 mL autoclave, which was placed in a furnace at 140 °C for 24 h, and then cooled down to room temperature. The obtained orange needle‐shaped crystals were washed with ethanol and dried in air. Due to their poorly diffracting nature (likely due to their pronounced 2‐dimensionality), a small batch of crystals were dissolved in 5 mL nitromethane (Sigma‐Aldrich), and the resulting bright red solution was filtered and placed for recrystallization at −10 °C. Strongly diffracting large block‐shaped single crystals of [PEA]_3_[Bi_2_I_9_] were formed over 48 h.

**TiO_2_ thin film*****via*****AACVD**: TiO_2_ thin films were deposited following literature procedure.^[90]^ 0.500 g of Ti(OEt)_4_ (0.46 mL, 2.20 mmol, Sigma‐Aldrich) was dissolved in 20 mL toluene (Fisher Scientific) to form the precursor solution. N_2_ was used as carrier gas with flow rate of 1.0 L/min. The glass substrate was kept at 450 °C during deposition time (20 min) and was subsequently let to cool down under N_2_ atmosphere. The transparent TiO_2_ films were extracted from the reactor at room temperature and stored individually until use.

**[PEA]_3_[Bi_2_I_9_] thin film*****via*****AACVD**: Thin films were deposited using a single‐inlet reactor described previously.^[91]^ The precursor solution was prepared dissolving 1.180 g of BiI_3_ (2.00 mmol, Sigma‐Aldrich) and 0.748 g of phenethylammonium iodide (3.00 mmol) in 5 mL anhydrous dimethylformamide (VWR) in a Schlenk flask equipped with a magnetic stirrer under N_2_ atmosphere, after which it was sonicated for 1 h. N_2_ was used as carrier gas for all depositions, with a flow rate of 0.8 L.min^−1^. Films were deposited on Pilkington® barrier glass substrates, TiO_2_‐coated barrier glass, and Pilkington® FTO substrates at 125 °C. Thin films were deposited over 45 min, after which substrates were cooled down under N_2_ flow. The resulting thin films were annealed and stored in atmosphere conditions.

**Characterization**: Single‐crystal X‐ray diffraction (SCXRD) datasets were collected by a SuperNova Atlas (Dual) diffractometer using Cu Kα radiation (λ=1.5418 Å). Gradient‐incident X‐ ray diffraction (GIXRD) patterns were collected over 4–45° (0.05° and 0.5 s per point) using a Bruker‐Axs D8 diffractometer with parallel beam optics and a PSD LynxEye silicon strip detector. This instrument used monochromatic Cu Kα_1_ radiation (λ=1.5406 Å) at 40 kV with 30 mA emission current, and the incident beam angle was set to 1°. The FT‐IR spectrum was measured in the range of 4000–400 nm^−1^ using a Bruker Alpha‐T ATR‐FTIR Fourier Transform Infrared Spectrometer. Film morphologies were studied using a JEOL JSM‐6301F field emission scanning electron microscope (SEM) and energy dispersive spectroscopy (EDS). X‐ray photoelectron spectroscopy (XPS) was performed using a Thermo Scientific K‐alpha spectrometer with monochromated Al Kα_1_ radiation (8.3418 Å), and a dual beam charge compensation system. Survey scans were collected in the range of 0–1200 eV at a pass energy of 50 eV. All peak positions were calibrated to adventitious carbon (284.8 eV) and plotted using CasaXPS® software. The surface roughness of films was characterized by atomic force microscopy (AFM) on a Keysight 5600LS scanning probe microscope taken at a scale of 20×20 μm^2^. Ultraviolet‐visible (UV‐Vis) spectroscopy was measured using a Shimadzu UV‐2700 spectrometer, and transmission/reflectance spectra were recorded in the 300–1100 nm range. Water contact angles (CA) were measured with an optical contact angle meter (FTA 1000) with a 5 μl water droplet under ambient environment. It was tested at three different positions on every sample for statistical comparison. Photoluminescence spectra were obtained by room temperature photoluminescence (PL) spectroscopy (Renishaw 1000) with a 325 nm He−Cd laser to investigate the optical properties of [PEA]_3_[Bi_2_I_9_] films.

## Conflict of interest

The authors declare no conflict of interest.

## Supporting information

As a service to our authors and readers, this journal provides supporting information supplied by the authors. Such materials are peer reviewed and may be re‐organized for online delivery, but are not copy‐edited or typeset. Technical support issues arising from supporting information (other than missing files) should be addressed to the authors.

Supplementary
