# Supplementary material for: Scalable Production of Ambient Stable Hybrid Bismuth‐Based Materials: AACVD of Phenethylammonium Bismuth Iodide Films
Source: Chemistry. 2021 May 27;27(36):9406–13. doi: 10.1002/chem.202100774 (PMC8361767; doi:10.1002/chem.202100774)
Supplement: Supplementary file 1 — Supplementary [file CHEM-27-9406-s001.pdf]

# Chemistry–A European Journal

Supporting Information


## Scalable Production of Ambient Stable Hybrid Bismuth-Based Materials: AACVD of Phenethylammonium Bismuth Iodide Films**


M. Wang, C. Sanchez-Perez, F. Habib, M. O. Blunt, and C. J. Carmalt*

## Author Contributions

M.W. Conceptualization:Equal; Data curation:Equal; Formal analysis:Equal; Funding acquisition:Equal; Methodology:Equal; Writing – original draft:Equal
C.S.-P. Conceptualization:Equal; Formal analysis:Equal; Methodology:Equal; Writing – original draft:Equal
F.H. Data curation:Supporting; Formal analysis:Supporting
M.B. Data curation:Supporting; Formal analysis:Supporting; Software:Supporting
C.C. Conceptualization:Equal; Funding acquisition:Equal; Project administration:Lead; Resources:Lead; Supervision:Lead; Writing – review & editing:Lead

# Electronic Supporting Information

| Empirical formula | $C_{24}H_{36}Bi_2I_9N_3$ |
|---|---|
| Formula weight | 1926.59 |
| Temperature/K | 293(2) |
| Crystal system | monoclinic |
| Space group | $P2_1/n$ |
| a/Å | 14.6383(3) |
| b/Å | 20.7493(3) |
| c/Å | 15.9278(3) |
| α/° | 90 |
| β/° | 113.193(2) |
| γ/° | 90 |
| Volume/Å$^3$ | 4446.85(15) |
| Z | 4 |
| $\rho_{calc}$/g cm$^{-3}$ | 2.873 |
| μ/mm$^{-1}$ | 64.549 |
| F (000) | 3364.0 |
| Radiation | CuKα (λ = 1.54184) |
| $2\theta$ range for data collection/° | 6.954 to 145.562 |
| Reflections collected | 31856 |
| Independent reflections | 8715 [Rint = 0.1255] |
| Goodness-of-fit on $F^2$ | 1.073 |
| Final R indexes [I>=2σ (I)] | $R_1$ = 0.1009, $wR_2$ = 0.2550 |
| Final R indexes [all data] | $R_1$ = 0.1108, $wR_2$ = 0.2762 |

$R_1 = (\sum ||Fo| - |Fc||/\sum |Fo|)$, $wR_2 = [\sum(w(F_O^2 - F_C^2)^2) / \sum(w|F_O^2|^2)]^{1/2}$

**Table S1**: Crystallographic data of $[PEA]_3[Bi_2I_9]$.

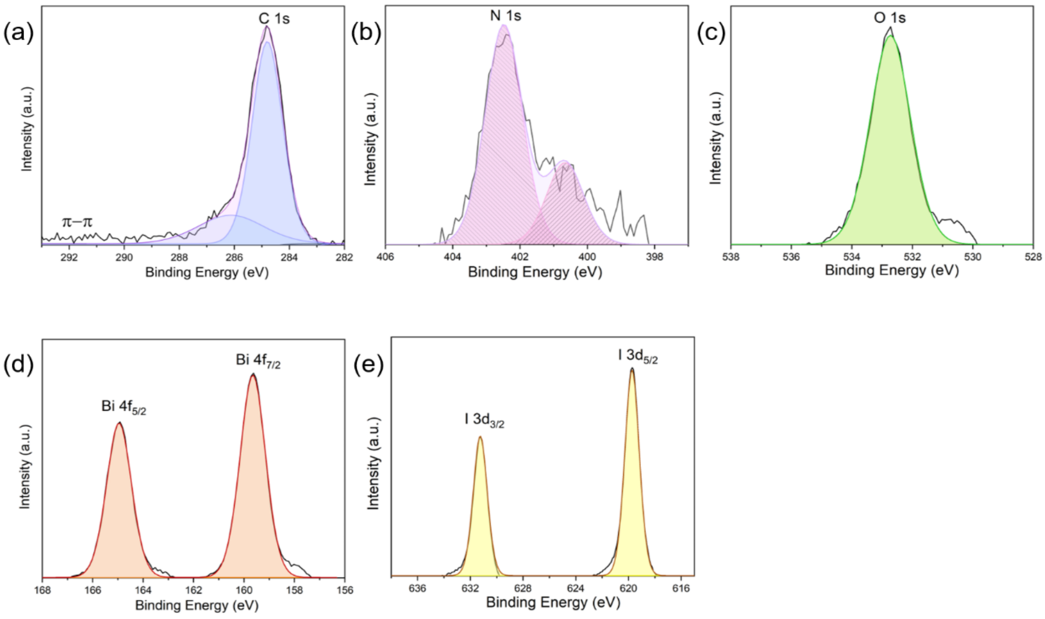


**Figure S1**: High resolution XPS spectra of a $[PEA]_3[Bi_2I_9]$ film deposited on glass substrate and annealed at 150°C in $N_2$: (a) C 1s, (b) N 1s, (c) O 1s, (d) Bi 4f and (e) I 3d.

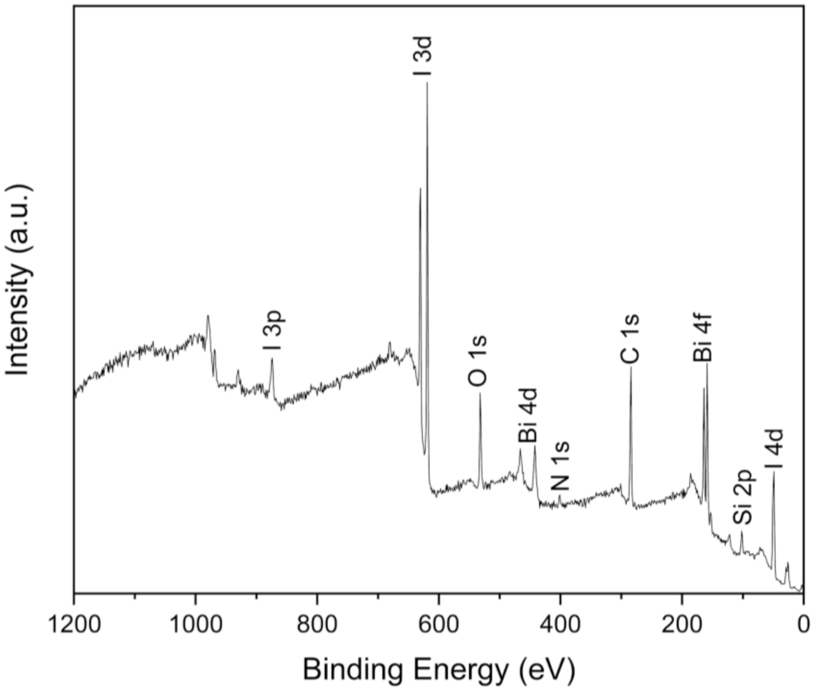


**Figure S2**: XPS survey spectrum of a $[PEA]_3[Bi_2I_9]$ film deposited on glass substrate and annealed at 150°C in $N_2$.

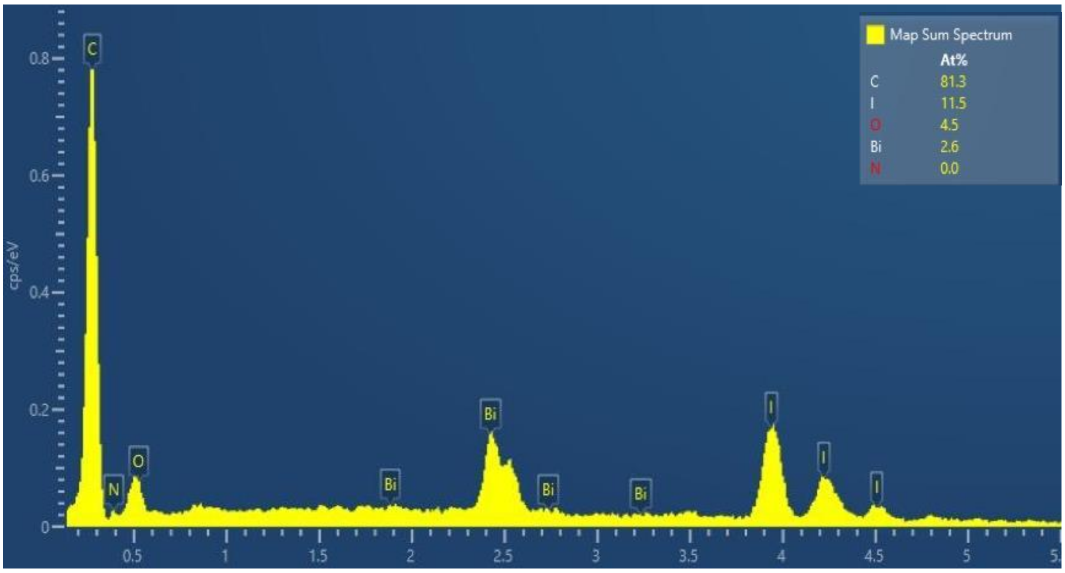


**Figure S3**: EDS analysis of $[PEA]_3[Bi_2I_9]$ thin film.

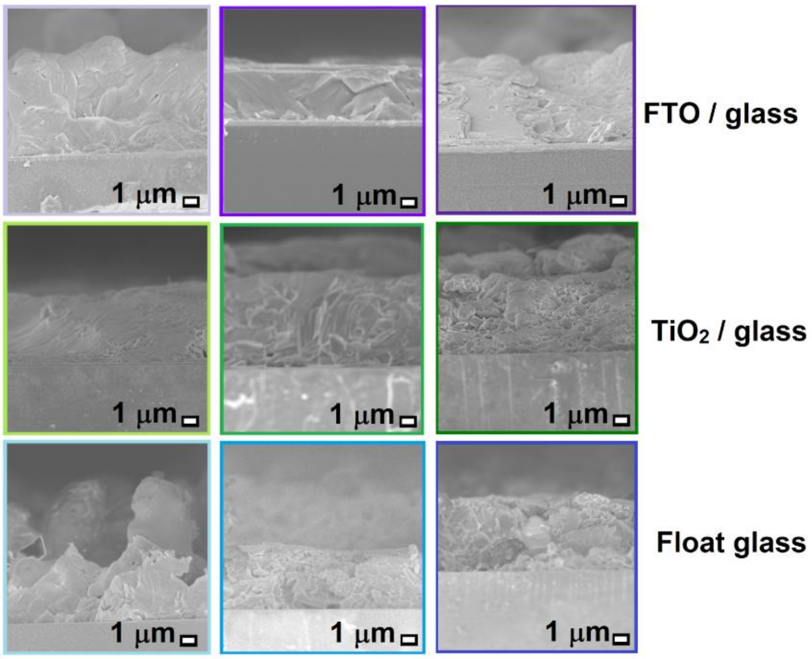


**Figure S4**. Cross-sectional SEM images of hybrid thin films as-deposited (left column), vacuum annealed at 120 ºC (middle column) and annealed in $N_2$ at 150 ºC (right column) on FTO/glass substrates (purple, a-c), $TiO_2$/glass (green, d-f) and float glass (blue, g-i). Magnification for all images is x 5000 and scale bar represents 1 µm.

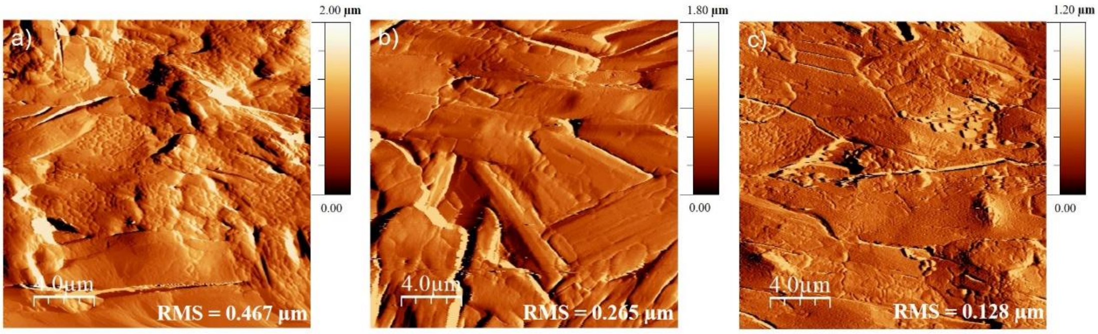


**Figure S5**. AFM images of $[PEA]_3[Bi_2I_9]$ thin films vacuum-annealed at 120 ˚C on (a) float glass, (b) $TiO_2$/glass and (c) FTO/glass. All images were of a 20 x 20 µm$^2$ area.

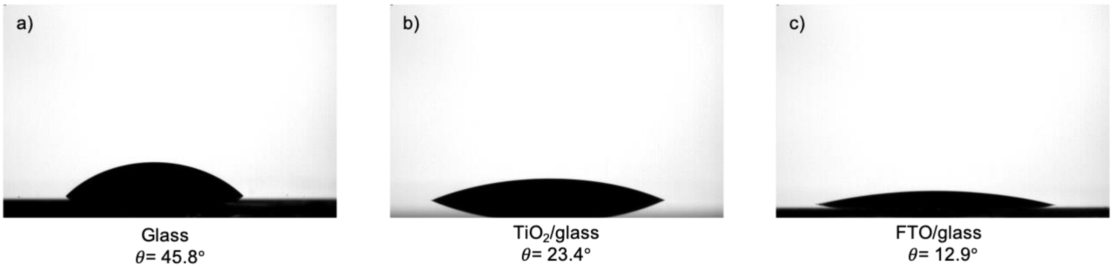


**Figure S6**: Images of DMF droplets on a) glass, (b) $TiO_2$/glass and (c) FTO/glass.
